# Current practices in spatial analysis of cancer data: mapping health statistics to inform policymakers and the public

**DOI:** 10.1186/1476-072X-5-49

**Published:** 2006-11-08

**Authors:** B Sue Bell, Richard E Hoskins, Linda Williams Pickle, Daniel Wartenberg

**Affiliations:** 1 Work conducted at the Division of Cancer Control and Population Sciences, National Cancer Institute, National Institutes of Health. Current address: U.S. Food and Drug Administration, 5600 Fishers Lane Rm 15-62 HFP-20, Rockville, MD 20857, USA; 2 Comprehensive Cancer Control Program, Washington State Department of Health,111 Israel Road, PO Box 47855, Olympia, WA 98504-7855, USA; 3 Division of Cancer Control and Population Sciences, National Cancer Institute, 6116 Executive Boulevard, Suite 504, Bethesda, MD 20892, USA; 4 Department of Environmental and Occupational Medicine, Robert Wood Johnson Medical School, University of Medicine and Dentistry of New Jersey, 170 Frelinghuysen Road, Piscataway, NJ 08854, USA

## Abstract

**Background:**

To communicate population-based cancer statistics, cancer researchers have a long tradition of presenting data in a spatial representation, or map. Historically, health data were presented in printed atlases in which the map producer selected the content and format. The availability of geographic information systems (GIS) with comprehensive mapping and spatial analysis capability for desktop and Internet mapping has greatly expanded the number of producers and consumers of health maps, including policymakers and the public.

Because health maps, particularly ones that show elevated cancer rates, historically have raised public concerns, it is essential that these maps be designed to be accurate, clear, and interpretable for the broad range of users who may view them. This article focuses on designing maps to communicate effectively. It is based on years of research into the use of health maps for communicating among public health researchers.

**Results:**

The basics for designing maps that communicate effectively are similar to the basics for any mode of communication. Tasks include deciding on the purpose, knowing the audience and its characteristics, choosing a media suitable for both the purpose and the audience, and finally testing the map design to ensure that it suits the purpose with the intended audience, and communicates accurately and effectively. Special considerations for health maps include ensuring confidentiality and reflecting the uncertainty of small area statistics. Statistical maps need to be based on sound practices and principles developed by the statistical and cartographic communities.

**Conclusion:**

The biggest challenge is to ensure that maps of health statistics inform without misinforming. Advances in the sciences of cartography, statistics, and visualization of spatial data are constantly expanding the toolkit available to mapmakers to meet this challenge. Asking potential users to answer questions or to talk about what they see is still the best way to evaluate the effectiveness of a specific map design.

## Background

Reporting spatial health statistics to policymakers and the public – either in a descriptive report or Web site application, or as part of the results of a carefully designed public health study – is challenging and sometimes daunting. Communicating conclusions and interpretations in a way that will inform without misleading the audience after conducting complex spatial analyses, applying sophisticated statistical methods (e.g., spatial smoothing), and using powerful information management technologies (e.g., geographic information systems), is an important and complicated, but manageable task if one pays careful attention to certain issues. The potential audience for the results of a spatial analysis of health data is no longer limited to scientists but now also includes the public, policymakers, the media, and a host of others. That is because health data are personal and confidential by their very nature, geography and maps introduced in elementary school are familiar tools used in daily life (e.g., weather maps, street maps, and atlases), and the Internet makes scientific data and results accessible to all.

This article provides a synopsis of some suggestions and comments by practitioners on how best to communicate the results of spatial analyses of health data. It draws upon the experience of designing and producing atlases for print and of providing interactive access to health statistics using the Internet. The article includes tips, information on risks, and special considerations for mapping health data from those who have faced the challenge of communicating public health information.

## Review

### Communicating effectively

Among the steps for the planning framework in *Communicating Public Health Information Effectively *[1] are (1) defining the purpose of the message, (2) identifying the audiences and their characteristics, (3) choosing the media, and (4) developing and testing the message. These same considerations apply to reporting the results of spatial analyses. The tools to communicate the message will usually be a map that may be accompanied by graphs or tables, and sometimes explanatory text.

Many people, even well-educated individuals such as physicians, have great difficulty fully understanding statistical information, due to their low numeracy skills [2-4]. Providing a clear context for statistical data through the use of examples, analogies, and diagrams has been shown to enhance understanding [5]. Providing audiences with results of spatial analyses through the judicious use of graphs, tables, and maps is also a useful approach for enhancing understanding of complex data sets.

MacEachren discusses how, through the abstract representations of maps, we can create knowledge as well as reveal knowledge [6]. With maps, there is not only the public representation with symbols to provide meaning but also a private, cognitive dimension. The map reader publicly focuses on the map's lexicon and function while privately using vision and cognition to perceive the map's meaning.

### Purpose of the maps

Three types of questions are generally asked of maps [7,8]. Consider a map of lung cancer mortality. The first type of question is a very specific rate readout task: What is the mortality rate in a certain area? Second, is a more general pattern recognition task: Are there geographic trends in the data, or regions of unusually high or low rates? The last is the most general map comparison task: Is the lung cancer mortality pattern similar to the pattern of smoking prevalence shown in a companion map?

The same map may not be equally suited to all of these questions. Environmental Systems Research Institute, Inc., (ESRI), a provider of GIS software, warns that "Trying to communicate too much in one map – having more than one purpose for the map – tends to blur the message and confuse the map reader. Using two or more maps, each focused on a single message, is always a better strategy" [9]. This philosophy is consistent with recommendations of Monmonier, who finds designing a map tailored to precise goals easier than forcing a single map to accommodate diverse objectives, and who recommends cartographic overlays for examining associations among two or more factors [10].

### Audiences and their characteristics

When providing statistical results to a general audience, presenting too much data or too many caveats can be counterproductive [[1] p. 43]. Most public audiences will not be familiar with statistical terminology but will respect the practitioner's background, experience, and expertise, and usually will assume the information is credible.

In contrast, scientific audiences and advocates involved in an adversarial situation often want details about the methodology used and information about the strengths and weaknesses of the specific analyses. The statistical analyst should specify when the presentation includes estimates, such as those from statistical models or smoothing, as opposed to direct observations.

To the extent possible, maps should be designed to stand alone when taken out of context. Titles should clearly state what data are being mapped. Citations for data sources and methods used should be provided. Map usability and interpretability should be tested on representatives from audiences likely to use the map. For example, prior to general release, individuals are sometimes recruited to answer questions based on the map. The questions should be designed to assess the map's clarity as well as the potential for misinterpretation.

### Making data accessible – static or interactive maps and databases

Information technology continues to change the landscape of what is possible in the display and communication of spatial data. In 1999 a special issue of the *Journal of Public Health Management and Practice *focused on the evolving role that GIS could play in public health [11]. In it, Richards et al. suggested that soon, "each community will have the capability to link together health information from a variety of different data sources and to recognize spatial data patterns that suggest where cost effective public health interventions can be applied" [11]. Much progress has been made since then, as illustrated by an extensive list of interactive Web modules [12].

**Figure 1:**
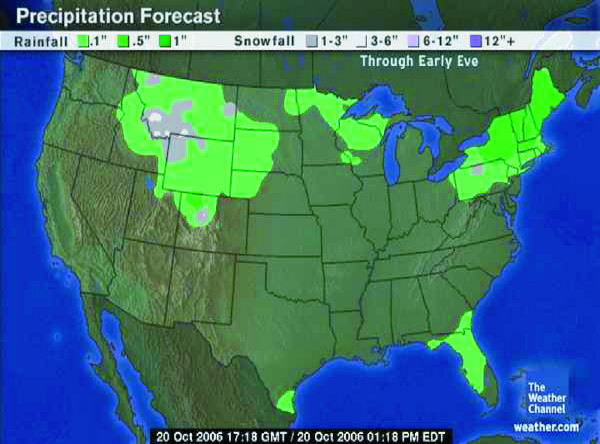
**Interactive weather maps**. Interactive weather maps [14] present precipitation forecasts that result from spatially smoothing reports from local monitoring stations and use color to indicate the type and quantity of precipitation.

Weather maps provide excellent examples of communicating spatial statistics using both static and interactive displays. Every major newspaper every day provides a static weather map. *USA Today *combines a national map with multiple small tables to provide local information consistent with Tufte's advocacy for multiwindow plots [13]. Figure 1 provides a screen image of a precipitation forecast from a popular Web venue for obtaining weather information [14]. Local reports of rain or snow accumulations are spatially smoothed and presented using colors to differentiate type and quantity of precipitation. These presentations of weather statistics have subtly educated the public on spatial probabilities and on statistical smoothing. Our challenge is to leverage the success of effectively communicating weather statistics to communicating health statistics.

**Figure 2:**
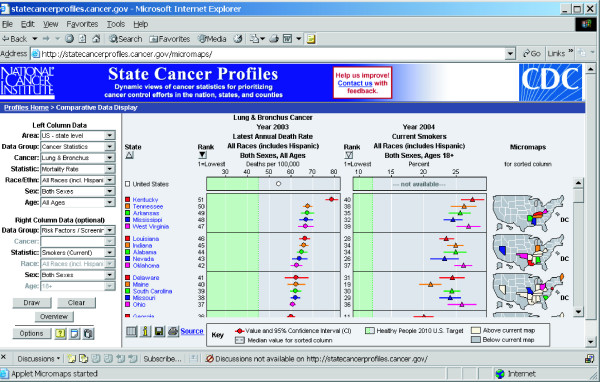
**Linked micromap (LM) plot**. Linked micromap plots [17] combine statistical graphs and maps into a single interactive graphic. The user chooses the statistics to display in the columns. The maps color the areas in order of the sorted column (indicated by the darkened triangle) in groups of five.

One approach, designed to support the program of Comprehensive Cancer Control planning for states and counties, led to a collaboration between the National Cancer Institute (NCI) and Centers for Disease Control and Prevention (CDC) that developed the State Cancer Profiles Web site [15]. That Web site links cancer statistics, screening and risk factor prevalence, and demographics to aid planners in focusing interventions on geographic areas and population subgroups that can most benefit. Figure 2 is an example of a creative display referred to as a linked micromap (LM) plot, which combines statistical graphs and maps by using the same colors to represent specific regions or features in all displays [16]. To explore cancer statistics using LM plots, users can select the link entitled "Comparative Data Display" on the State Cancer Profiles Home page [15] or by using a direct link [17]. The user controls the data elements shown and level of geography using the pick lists on the left. The authors encourage the reader to access the LM plots Web page and to explore the interactive features. With most Web browsers the LM plots will work on the first try; for access problems, refer to the frequently asked questions (FAQ) link at the foot of the Web page for explanations and instructions.

From the display in Figure 2, one can see that states with high lung cancer mortality rates also have a high prevalence of current smokers and that these states are clustered in the Southeast. Note that inferences based on comparisons of aggregate or grouped data, such as state rates, are subject to a situation known as the ecologic fallacy [18,19]. That is, associations observed at an aggregate level may be inconsistent with associations observed among individuals. In the simplest terms, while we may know that smoking rates and lung cancer rates both are high in a given state, we do not know if those who died of lung cancer were the smokers.

Changes in the production of atlases have also produced new analytic and communication opportunities. Historically, atlases were designed as books. However, over the last 10 years, mapping of health data has progressed from static maps designed for print media where the author selected both data and layout, to dynamic, interactive mapping over the Internet where the public may produce maps for their own purposes. The most recent edition of *The Atlas of United States Mortality *[20] was designed for print release but was also released on the Internet as an Adobe portable document format (PDF) file. Likewise, other recent health atlases that were designed for print release were made accessible on the Internet as PDF files. Examples include atlases on heart disease and on stroke [21,22] as well as *Mapping Census 2000: The Geography of U.S. Diversity *[23]. NCI's *Atlas of Cancer Mortality in the United States: 1950–1994 *[24] was also published first as a book and then released on the Internet as a PDF file. However, NCI went further, expanding the Web site [25] to provide interactive mapping, animation of maps over time, and statistical graphs of the cancer mortality statistics.

**Figure 3:**
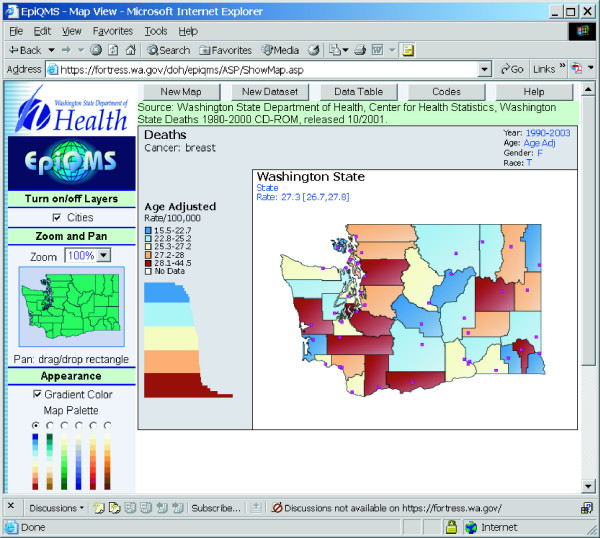
**Epidemiologic Query and Mapping System (EpiQMS)**. The Washington State Department of Health has developed EpiQMS [26], which combines maps, graphs, and tables for mortality statistics and population statistics. The points represent major cities. EpiQMS is also used by the Pennsylvania Department of Health.

**Figure 4:**
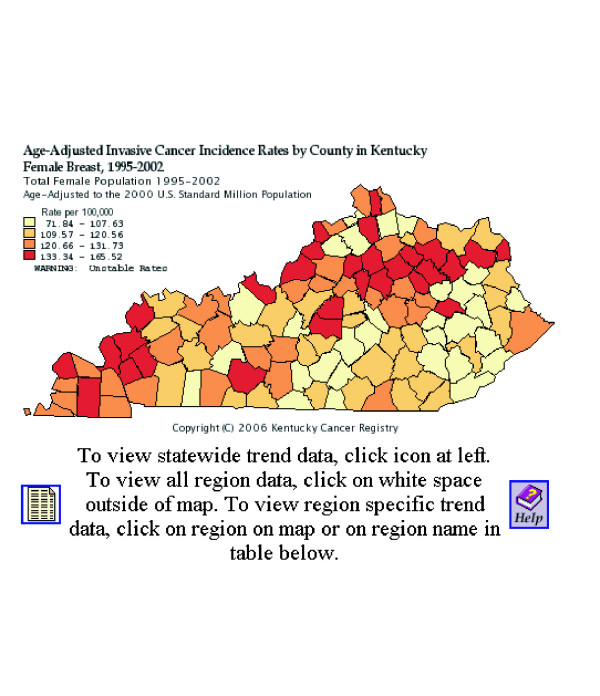
**Kentucky Cancer Registry's interactive query and mapping**. The Kentucky Cancer Registry provides user-controlled queries and maps for cancer incidence and mortality data [28].

**Figure 5:**
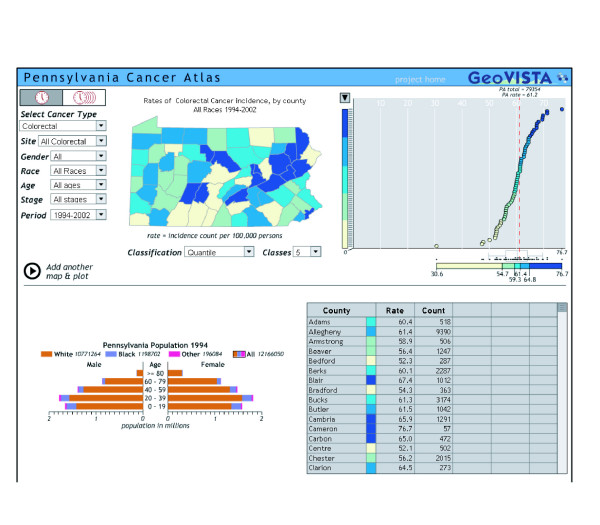
**Pennsylvania Cancer Atlas's dynamic linked maps, tables, and graphs**. The Pennsylvania Cancer Atlas provides dynamic links for counties selected on either the map, table, or graph [33,76].

Finally, some health data repositories are exploring ways to make their data more accessible via the Internet. Many state health departments and state cancer registries provide public access to their health statistics. The State of Washington has developed EpiQMS (Epidemiologic Query and Mapping System), shown in Figure 3, which combines maps, graphs, and tables for vital statistics data [26]. The State of Pennsylvania has also implemented EpiQMS [27]. Similarly, the State of Kentucky's cancer registry provides interactive access to its cancer statistics, as shown in Figure 4[28]. Some state cancer registries also regularly publish static maps and tables to report progress. Geospatial One-Stop [29] is a U.S. government initiative to promote the sharing of geo-referenced data. The National Science Foundation's Digital Government/Quality Graphics initiative [30] has promoted creative data displays such as the linking of maps and statistical time-series plots in the Exploratory Spatio-Temporal Analysis Toolkit, developed by the Pennsylvania State University's GeoVista Center in collaboration with NCI [31,32]. The Pennsylvania Cancer Atlas previews this next-generation technology based on the GeoVista research to provide dynamic links between maps, tables, and graphs, as shown in Figure 5[33].

### Guidelines for communicating spatial information

When presenting the results of statistical analyses of spatial data, choose a medium that best suits the message and the needs of the audience. Where control of the message is important, static maps will continue to be the most effective, although good tables, graphs, and explanatory text are still needed in order to ensure that different people will see the same thing in the maps. For example, camera-ready maps depicting the spreading epidemic of obesity in the United States were included in an article in the *Journal of the American Medical Association *[34], and this led to front-page newspaper and national broadcast news coverage [[35] p. 86]. Further, the maps spurred debate regarding which was the greater public health problem, smoking or obesity.

Alternatively, interactive access to data collected by cancer registries or health departments promotes public interest and exploration. In the short term, however, this broader use of the data may increase the risk of misuse or misinterpretation due to users' inexperience. Nonetheless, interactivity may be the attribute of scientific communication with the greatest potential for increasing understanding of complex health information and influencing audiences, especially when available online [35-38]. The importance of interactivity is related to the greater value of participation in both the process and content of communication. Research shows that when audience members are involved in the design and dissemination of health communication, the results and messages reported are more likely to be accepted by the broader audience [35,36]. Interactive access to data promotes audience involvement and provides opportunities for feedback and exploration of data sets. GIS technology can enable a public health practitioner to explore areas of concern interactively with an audience. Reference layers can be added in real time to base layers to allow concerned citizens to consider broader contexts. The geographic context allows citizens to identify their neighborhood and see how it compares with other similar neighborhoods.

### Developing and testing the map

Maps should be produced using sound cartographic principles and then be tested on representatives from potential audiences, including the public. This guidance applies both to static maps and interactive Web sites. Because interactive Web sites potentially provide more options and combinations for the user to select data to map and to customize the map for presentation, more testing is involved.

There are many excellent cartography textbooks to help non-experts learn how to create accurate, clear, and attractive maps (e.g., Robinson et al.'s *Elements of Cartography *[39] or Slocum et al.'s *Thematic Cartography and Geographic Visualization *[40]). If possible, when constructing a map, those with limited experience should consult with a cartographer on map design or have a cartographer review a draft map design; however, excellent guides exist for GIS users [41-43]. A work group of the North American Association of Central Cancer Registries (NAACCR) [44] published *Using Geographic Information Systems Technology in the Collection, Analysis, and Presentation of Cancer Registry Data: A Handbook of Basic Practices*, which includes a section on cartography [45]. The complete handbook is available for download from the NAACCR Web site.

Some key points from the handbook's section on cartography include the map layout, statistical maps, colors, and testing the map design. Special considerations include the limitations of the data, the limitations of the analysis, confidentiality, uncertainty in estimates, and potential misinterpretation of results.

#### Map layout

In designing a map layout, common elements usually include the following.

• The title matches the theme and audience, is concise but accurate, and is simple while clearly indicating the purpose of the map.

• The legend provides for symbol interpretation, is designed with ease of interpretation and clarity in mind, and includes any map features that might be unknown to the audience or might otherwise cause confusion.

• The map body includes the necessary amount of data and detail while recognizing that too much detail can result in losing the intended message.

• The scale of true distance to map units is a representative fraction (e.g., 1:24,000-one centimeter on the map is equivalent to 24,000 centimeters on the ground) or a graphic of the distance measure. A small-scale map shows a large geographic area so things look small, while a large-scale map shows a small geographic area so things look large.

• The direction indicator, such as a north arrow, orients viewers who are unfamiliar with the area portrayed.

• Labels for place names or data values are included only as needed, considering the major communication goal of the map. For example, major cities in a state may be shown to provide reference points.

• The source should provide clear reference links to data sources available for map user follow-up.

Optional map elements include:

• Projection of the map that was used to transform latitude and longitude locations to x, y coordinates. The projection process flattens the earth's curved surface, creating distortions in area, distance, direction, or shape. Usually a map of the continental United States will use the Albers Equal-Area map projection, which preserves area (i.e., any area defined on the map, such as 1-inch square area that is 1% of the total map surface, corresponds directly to the same proportion, e.g., 1%, of the true surface being mapped). When using multiple GIS map data layers, each map layer must use the same projection and scale so that map features align properly when overlain.

• Cartographer's name or organization.

• Date of production (this is especially important for time-sensitive data).

• Neat line around the map extent, which indicates exactly where the map begins and ends.

• Locator maps (maps of large geographic regions that include the region of interest to indicate exactly where the map or feature of interest begins and ends).

• Inset map (large-scale map of a zoomed-in portion of the main map).

• Index maps (these depict the location of each of several map compositions that comprise coverage of an area).

Consider the map's purpose and whether each map element is necessary for accurate interpretation of the map by the map reader. Ensure that the layout focuses on the most important feature of the map and not on a background element.

#### Statistical maps

Map types commonly used for health statistics include:

• Classed choropleth maps, which shade each area based upon its classification into a set of categories and support rate look-up and pattern recognition.

• Isopleth maps, which use contours to show patterns. These are commonly used for measures that are continuous over space, such as elevation or temperature. They are appropriate for representing disease rates or spatially smoothed rates. A smoothed map is a map that has removed some random variation in the underlying rates, e.g., by a spatial moving average that borrows information from neighboring areas or from regions with more stable rates.

• Graduated or proportional symbols, where the size of the symbol is proportional to each mapped value or to a representative value of each rate category. An example would be a bar chart over each census tract that depicts the percentage population distribution of racial groups but with the size of the bar chart proportional to the total population.

• Area symbols, which are used to represent nominal or qualitative data that in concept or in fact extend over an area. For example, an area symbol could depict land use.

For classed choropleth maps, equal interval classification is useful when the mapped quantity is in familiar units (e.g., packs of cigarettes smoked per day). However, for adjusted rates that are only meaningful in relation to other similarly adjusted rates, Brewer and Pickle [46] conducted a study in which subjects evaluated seven potential classification methods for conveying patterns of mapped rates and found that the quantile method was best. The quantile method, also known as the percentile method, ranks the enumeration units by the variable of interest and then places an equal number of enumeration units into each class. The quantile classification tested used quintiles, or five classes, so 20 percent of the units were placed in each class by rank. Quintiles and quartiles are common choices for quantile classification.

When preparing a series of maps, such as is done for animating maps over time, the same classification method and values should be used for each map in the series for consistency [46]. Usually the classification range is constructed from the midpoint in the time series and then applied to each map in the series.

#### Colors

Selected colors should not violate generally accepted conventions. For example, individuals are accustomed to blue representing water and green representing vegetation. The convention for quantitative data is that either darker or warmer colors represent higher values. For example, the historical use of reds for high rates and blues for low rates in cancer mortality maps sets a strong expectation. When data are classified into groups (classes), colors need to be assigned that work well in distinguishing between the classes. Recent National Science Foundation-funded research by Cynthia Brewer has produced a Web site that is particularly useful for making the color choices for sequential (light to dark); diverging (dark to light of one color, then light to dark of another color); and qualitative color schemes [47]. Diverging schemes are useful when one of the goals of the map is to show where rates are higher or lower than some middle value (e.g., U.S. overall rate). The Web site also helps the map designer to choose appropriate colors for use by the color blind (most commonly those readers who have a particular problem distinguishing red and green), for printing in black and white and for displaying on a laptop computer or a projection system.

### Testing the map design

All map designs should be tested to ensure that they communicate the intended message with the intended audience. The investment in testing should be proportionate to the consequences of misinterpretation. Testing of the design can range from a simple walk-through with a peer for maps intended for internal communication only, to more thorough usability testing with representatives of the target audience for maps expected to have broad distribution.

The first stop in validating a map design should be with a peer who is familiar with maps commonly used in the subject field. In preparation, develop questions that a map reader should be able to answer, and consider the ways in which the answers should be consistent with the messages that the map is to convey. If available, a cartographer should also be consulted at this time.

After incorporating the suggestions made by peer reviewers and/or by the cartographer, the same questions can be used to test the maps on several people who are representative of potential audiences. If the public will be using the maps, it is very important that selected representatives be among those tested. Family and friends are a convenient source of informal test subjects, but one must also seek reviews from members of the special interest groups likely to use the maps.

The formality and extent of the testing will depend upon the sensitivity of the data being presented, the potential impact of misinterpretation, and the potential breadth of the audience. Many Web sites go through formal usability testing [48]. When a formal usability test is warranted, scenarios are developed to guide and focus the user's exploration of features and content in order to elicit the most information about areas of particular concern. The test facilitator should be both independent of the project development team and experienced in conducting usability tests. It must be made clear to the user volunteering for the test that this is a test of the map's ability to communicate and *not *of the users themselves. Focus groups can provide a qualitative evaluation of a map's effectiveness and can be used to elicit suggestions for further development. At a minimum, informally ask one or more individuals who are not close to the research to review the maps and to answer the questions and describe aloud what the maps communicate to them.

### Special considerations

There are special considerations in communicating the results of a spatial analysis of health statistics that are not issues for other spatial applications, such as weather statistics. Consideration must be given to the following: limitations of the data, including its quality; limitations of the analysis; confidentiality; uncertainty in the estimates; effects of data smoothing; and misinterpretation of results.

#### Limitations of data

Spatial health data has unique characteristics. The article by Boscoe et al. [49] discusses current practices in spatial analysis of major types and sources of data, including cancer registries, population data, health surveys, environmental data, and remote sensing data. In order for policymakers and the public to consider the results trustworthy, information must be included on the source and quality of the data. Metadata for the data source should include some indication of the data quality. The Federal Geographic Data Committee [50] provides information on metadata standards for the United States. Quality of geocoding can be problematic, so a definition and measure of geocoding accuracy and success is needed. Disease classification and cause of death classification can be open to interpretation. Cancer consists of many diseases with different etiologies, so cancers should only be grouped when it makes biological sense to group them. For survey data, sample size and response rates are important information to include as indications of quality and reliability. Unfortunately, data needed for an analysis such as residential history and measures of exposure are often unavailable. When an analysis proceeds with what data are available, it is important that results discuss any assumptions and any limitations of the data.

#### Limitations of analysis

Jacquez's article on "flies in the ointment" [51] and Anselin's "How (not) to Lie with Spatial Statistics" [52] discuss in detail the limitations of spatial analyses of health data. Quantitatively powerful techniques are available for identifying locations of potential clusters, hot spots, cool spots, etc [53-55]. However, the inferences that can be drawn are often limited, because clustering does not necessarily illuminate the etiology – especially since scant information may be available with respect to an individual's exposure history to possible putative agents. There are often spatial and temporal mismatches, where information on cases and exposures do not align in space or time. This is particularly a problem with cancer's long latency (lag time) from potential exposure to diagnosis. People move around over time, both during a single day and over a period of years. Cases in a geographic area may have been exposed elsewhere, or people exposed may have relocated. The ecologic fallacy (i.e., that associations observed at an aggregate level may be inconsistent with associations observed on individuals) is inherent in most spatial analyses of grouped health data [18]. In addition, the level of spatial aggregation can affect the results; e.g., a multi-state, regional analysis of small area data may lead to different conclusions than analysis of each individual state [56]. As with limitations on data, it is important that a supporting section discuss limitations of the analysis.

#### Confidentiality

Public health reporting systems and cancer registries were committed to the protection of the privacy of the individual even before the mandates included in the Health Insurance Portability and Privacy Act. There is a natural tension between providing information useful for local action and ensuring confidentiality of sensitive personal health data [57]. Methods that have been used to protect confidentiality include the following: (1) spatial and temporal aggregation, (2) adding geographic or etiologic context variables to original unmasked data and then removing the geographic identifiers, (3) random small-scale relocation of individual records, and (4) limiting access to potentially identifiable data through a user- and/or function-restricted computer environment.

First, aggregation over space and time has been used historically for health statistics as one way to ensure confidentiality. For example, CDC's WONDER system [58] will only provide mortality rates for counties in the United States with populations less than 100,000 persons when the data has been aggregated over at least 3 years. Another common constraint is to set a threshold value such as requiring an aggregation of 5 or more cases before counts or rates can be released for a geographic area. This spatial aggregation leads to health statistics often being grouped when reported in tables and then mapped using choropleth or area-shaded maps. Aggregation, however, limits the resolution of the data and thus can limit interpretability, increase the possibility of bias due to the merging of heterogeneous data, and greatly affect or prevent the typical adjustments for bias, confounding, and effect modification. Bias is an error that can occur based on the collection or analysis of data such as under- or over-reporting the number of cases over time or in a subgroup. Confounding occurs when a variable is related to both the exposure and the disease in such a way that the apparent association between them is altered. Age is the most common confounding variable in health data and has led to the practice of mapping age-adjusted rates for use by epidemiologists. An effect modification occurs when the relationship between disease and exposure is different for different levels of a confounding variable.

Second, geographic or etiologic context variables can be added to original unmasked data for a public use or research data set, and then the geographic identifier can be removed. In this approach, the individual records are geocoded to an administrative unit such as a census tract and then some attributes of interest from that census tract are associated with the individual record. Care must be exercised to ensure that some combination of the contextual variables does not serve as a geographic identifier of an individual. This approach can be particularly useful where geography is already serving as a surrogate for these contextual variables. For example, much research into health disparities focuses on socioeconomic factors. The spatial location of an individual is not so much of interest as that the individual lives in a high-poverty area or an area of high air pollution, or draws drinking water from a contaminated source.

Third, the location of individual records on a map can be relocated randomly [59]. Points are shown, but the locations have been moved a random distance and a random angle from their original source, giving a general picture of the spatial distribution of the data without allowing for identification of the individuals. In the investigation of cancer clusters, there is pressure to show the true location of the subject, but this usually cannot be done without the written informed consent of every subject.

Finally, access to identifiable data may be limited to a controlled research environment, although this is not typically a problem in health departments carrying out surveillance or cluster investigations. Researchers with protocols approved by an institutional review board (IRB) sometimes can work with the identifiable data but may be restricted to publishing results in formats that protect the confidentiality of the subjects. CDC's National Center for Health Statistics provides such a facility for researchers to work with individual respondent data from their national surveys [60]. The Long Island Breast Cancer Study Project Geographic Information System provides public access to limited data but is primarily intended as a tool for researchers with IRB approval to study relationships between environmental exposures and breast cancer [61].

Rules for accessing geocoded data vary greatly. Some states currently preclude researchers from working with data geocoded to the residential street address, while others encourage such usage.

#### Uncertainty in estimates

Extreme observed rates are often based on the fewest observations and are therefore unstable, or highly variable, estimates of the true rate. Approaches that have been used to address this issue include spatial smoothing [62] or spatial filtering [63] of rates, hatching areas with unstable rates [64], suppression or blanking out of unstable rates [24,65-67], or use of two or more maps where the first map is of the observed rates and the second map is of some measure of variability (e.g., residuals, standard deviations, or statistical significance) [64]. A statistical significance test can be added to the level of the rate in the classification for inclusion in the legend [24]. In Figure 2, confidence bars show uncertainty in rates in an LM plot [68,69] used in the State Cancer Profiles Web site [15].

Audience, media, and purpose of the map all influence which approach can and should be used. The public is probably not familiar with statistical variability and testing and would be confused by the presentation of two or more maps as commonly used for scientific audiences. Nevertheless, the public is familiar with a weather map that has spatially smoothed temperatures to show the weather pattern and that uses a color scheme of warm colors for warmer temperatures and cool colors for cooler temperatures. Static maps in print or provided over the Internet can easily provide spatially smoothed or spatially filtered data to support pattern or cluster detection. EpiQMS [26,27,70] has calculated spatially smoothed rates for counties in Washington that can then be mapped interactively as an area-shaded map, as shown in Figure 3. When the primary purpose is to provide rate read-out functionality, the LM plot presented in Figure 2 provides a combination graph and map format, where the graph includes a confidence interval for the rate estimates while also providing an area-shaded map of the observed rate.

There are three alternatives for indicating unreliable rates in an area-shaded map: hatching, use of less saturated colors, and use of a neutral color such as light gray. Hatching was used for the U.S. Mortality Atlas [64] to indicate unreliable rates. Depending upon the mapping software, however, hatching can be problematic to implement. When the areal unit is small, it may be difficult to see what is and what is not hatched. When hatching is not practical, one of the two color options is usually used. When it is possible to use very saturated colors, then the use of less saturated colors for less reliable rates retains for the map reader the basic information on the level of the rate. Several recent health atlases [24,65-67] have suppressed unreliable rates by displaying those regions using a neutral color. In tests conducted during the development of the U.S. Mortality Atlas [20,64,71], it was shown that both blanking unreliable areas and reducing color saturation impair cluster identification but that indicating unreliability by hatching or by using separate maps for rates and reliability worked well for cluster identification [64]. Suppressing an area's rate can be frustrating for users of the map and, perhaps more troubling, can make the public suspicious that information is being withheld. In addition, hatching or suppression approaches are limited, because they display only a binary assessment of reliability rather than provide a continuous measure of the degree of reliability as can be shown with two maps.

#### Effects of data smoothing

Data smoothing provides a picture that presents broad patterns, as can be seen in the weather map in Figure 1, but it can remove detail from the map that would permit reading an original value from a specific place. An underlying assumption is that areas in close proximity will be more alike, but this is not always true. Instead, it may be desirable to borrow strength from areas with similar demographics. A further complication is that simple unweighted smoothing will treat all rates as equally reliable, possibly smoothing away important and reliable "hot spots" of high rates. To illustrate, HIV mortality rates are higher in cities, where rates are based on large numbers of cases, compared with rates in surrounding suburban or rural areas with smaller populations. Unweighted smoothing of these rates will remove the isolated urban "hot spots," whereas smoothing HIV rates weighted by their population or other measure of reliability will retain the reliable high city rates while smoothing rates in less populous places to be more like neighboring areas [62].

Smoothing of observed rates can also be accomplished by regression modeling of the underlying data. For example, statistical models of the associations between cancer incidence rates in a subset of U.S. states and a number of sociodemographic factors have been used to predict cancer incidence across the entire United States [72]. These predictions are statistically smoothed compared to the original observations.

In addition to illustrating broad patterns in the data, smoothed rate maps can help to remove the dependence of apparent spatial patterns on artificial administrative boundaries by smoothing the patterns across these boundaries (see, for example, maps developed for community planners in Iowa [73]). A number of smoothing methods are available; a method needs to be chosen that ensures that features of interest to the reader are not lost.

#### Misinterpretation of results

In an editorial discussing the "promise and pitfalls" of GIS technology [18], Melnick and Fleming note that integrating complex data into an easy-to-understand picture could lead to misunderstanding and misuse. There is the temptation to infer causation from correlation and to make inferences about individuals from population data (a.k.a. the ecologic fallacy) [74]. To minimize this risk, it is important that maps be tested on a representative audience, as discussed above.

When the intent of the maps is to inform and educate the public about cancer risk, it is important that the provider of the information be informed about risk communication. Risk perception combines the perceived probability or likelihood of an event and the severity of the consequences of the event. To the public, even one case of childhood brain cancer in the neighborhood elevates concern by combining an event with severe consequences and making it highly probable because it is someone they know. Discussion of the absence of a "statistical excess" of childhood brain cancers in the area is likely to increase mistrust and concern that the truth is being hidden. Ratzan et al. [75] provide worksheets for planning risk communication to ensure that the messenger is prepared to address the public's concerns. Use of interactive maps can help involve the public in exploring the data spatially and increase their understanding of the complexity of assessing the risk of a potential exposure.

## Conclusion

Lessons learned in developing effective communications media should be applied to communicating results of spatial analyses of health statistics. Essential steps include defining the purpose of the communication, identifying potential audiences and their characteristics and needs, choosing the media, and testing the delivery on representatives from the audiences to ensure effective communication is possible. In particular, consider the numeracy skills of the audiences and their need for the information, and present the data appropriately.

Desktop geographic information systems and interactive mapping capabilities on the Internet have put the power of communicating spatially into the hands of the public. However, most users of these technologies have not been trained in either cartography or statistics. Developers of quantitative mapping systems should ensure that default settings on their software or applications are based on sound cartographic and statistical principles. Users of these systems who publish maps should always test their map designs on potential consumers to ensure that the maps are communicating without misinforming. For statistical maps, consider map types beyond the traditional classed choropleth map that shades each area. Isopleth maps effectively show patterns and are well understood by the public who view weather maps daily.

Mapmakers should ask, "What is the message?" and "How will the message be used?" Then, they should choose a map style that will communicate that message to the target audience. Finally, mapmakers should test that the map audience understands the intended message.

## Abbreviations

CDC – Centers for Disease Control and Prevention

EpiQMS – Epidemiologic Query and Mapping System

GIS – geographic information system

IRB – institutional review board

LM plot – linked micromap Plot

NAACCR – North American Association of Central Cancer Registries

NCI – National Cancer Institute

PDF – portable document format

U.S. – United States

## Competing interests

The author(s) declare that they have no competing interests.

## Authors' contributions

BSB led the effort to draft the manuscript, drawing upon the subject matter expertise of REH and DW. REH finalized the manuscript, incorporating feedback from peer reviewers and updating to keep abreast of changes in the field. LWP convened the panel of experts that motivated this manuscript and also guided the manuscript's development. BSB, REH, LWP, and DW read and approved the final manuscript.
